# Recent achievements of the National Center Biobank Network (NCBN) bioresources through Japanese fiscal year 2025

**DOI:** 10.3389/fgene.2026.1910504

**Published:** 2026-10-19

**Authors:** Yosuke Omae, Katsushi Tokunaga

**Affiliations:** 1 Central Biobank, National Center Biobank Network (NCBN), Tokyo, Japan; 2 Genome Medical Science Project, National Institute of Global Health and Medicine (NIGHM), Japan Institute for Health Security (JIHS), Tokyo, Japan

**Keywords:** biobank, clinical information, database, genomic data, human biospecimens, participants, samples of diseases, whole-genome sequence

## Abstract

The National Center Biobank Network (NCBN) is constituted by a network of biobanks from five National Centers and the Japan Institute for Health Security (JIHS). The NCBN primarily collects samples from patients with various diseases, amassing a total of more than 250,000 participants and 800,000 human biospecimens as of the end of March 2026. The primary sample types are blood and DNA, with spinal fluid and frozen tissue samples also collected depending on the characteristics of the disease. These samples are accompanied by highly specialized and traceable clinical information supported by advanced medical care and expertise. Moreover, some samples are accompanied by whole-genome sequencing data. Consequently, these resources provide a unique platform for translational research, biomarker discovery, and genomic medicine in Japan. In the present review, we provide a detailed account of the recent achievements of NCBN biobanks and their biospecimens and genomic data.

## 1 Introduction

Human-derived samples and clinical data are essential for medical research. Biobanks are facilities that store and provide these resources widely. The National Center Biobank Network (NCBN) is a networked and federated organization of the six biobanks operated by National Centers (NCs) for advanced specialized medicine in Japan, which are supported by the Ministry of Health, Labour and Welfare of Japan (Omae et al., 2022). Of the 6 NCs in Japan, four are located in the Tokyo metropolitan area, while the remaining two are located in the Osaka and Aichi areas. Each NC has a specialized hospital, research institute, and biobank to conduct basic and clinical research on major diseases that have a significant impact on national health in Japan. Each NC biobank has established a “disease-based biobank” where disease-specific biospecimens and clinical information are collected.

The Central Biobank of the NCBN, which serves as the administrative and data management hub of NCBN, has been promoting public relations activities to utilize patient-derived bioresources for medical and research purposes since 2011. The NCBN has created websites, newsletters, and leaflets, developed a shared catalogue database (DB) that enables searches for bioresources from the 6 NCs, and provided one-stop services for individuals and organizations seeking to use these bioresources. To this end, the NCBN has facilitated discussions on data sharing within the network of these 6 NCs and advanced the standardization and unification of sample collection and provision methods to promote drug discovery and biomarker development. In April 2025, the National Center for Global Health and Medicine (NCGM), a constituent organization of the NCBN, merged with the National Institute of Infectious Diseases to form the Japan Institute for Health Security, (JIHS) (Kokudo et al., 2025). As a result, both the Central Biobank Secretariat and NCGM Biobank have moved to the JIHS, creating a new structure that includes the JIHS and 5 NCs: the National Cancer Center (NCC), National Cerebral and Cardiovascular Center (NCVC), National Center of Neurology and Psychiatry (NCNP), National Center for Child Health and Development (NCCHD), and National Center for Geriatrics and Gerontology (NCGG). However, NCBN activities continue as before (Figure 1).

**FIGURE 1:**
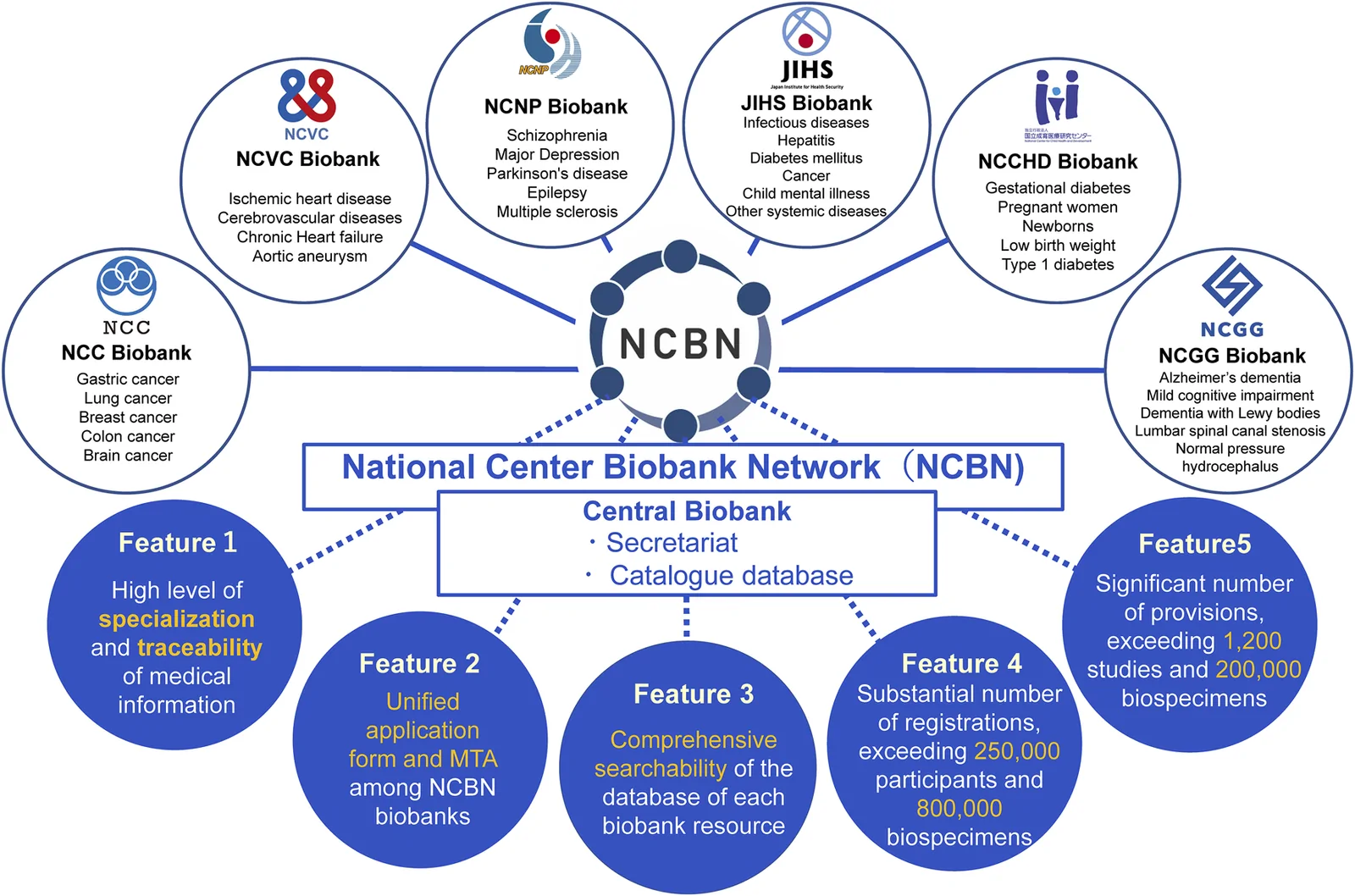
Overview of the NCBN. The NCBN has been constructing a networked and federated organization of biobanks in five National Centers and the JIHS to utilize patient-derived bioresources for medical and research purposes through the Central Biobank.

By utilizing human biobanks, researchers can not only elucidate the causes of diseases, but also advance basic research such as pathophysiological analysis. Furthermore, researchers can expand these efforts into clinical trials that apply these findings to clinical settings, potentially delivering new medical treatments to the public more quickly and at a lower cost, thereby helping to curb future increases in healthcare costs. In particular, the use of genomic information in medical care and research for the development of recent treatments and preventive measures has advanced significantly, and the critical importance of sample quality has been recognized globally. In response to this trend, the International Organization for Standardization (ISO), headquartered in Geneva, Switzerland, published the first edition of the international standard for biobanks, ISO 20387, in 2018 (International Organization for Standardization, 2018). In Japan, the Japan Accreditation Board (JAB), a public interest incorporated foundation, began accrediting biobanks in 2023 based on the Japanese Industrial Standard JIS Q 20387 (General Requirements for Biobanking), which was developed based on the international standard ISO 20387 (Japan Accreditation Board, 2026). In March 2024, four biobanks in Japan, including the NCGG and NCNP biobanks in the NCBN, received accreditation for the first time, demonstrating a commitment to align with global trends. Furthermore, the expansion of this initiative to the remaining NCBN biobanks is under consideration. As follows, we provide a status update of NCBN bioresources and their representative achievements.

## 2 Recent achievements of the NCBN

### 2.1 Overview of bioresources in NCBN biobanks

As a hospital-affiliated disease biobank, the total number of registered individuals and samples across the six NCBN biobanks has continued to grow, exceeding 250,000 registered individuals and 800,000 registered samples in fiscal year 2025 (Figures 2A,B). The Central Biobank of the NCBN collects and publishes bioresource information in a centralized platform called the NCBN Catalogue Database (https://www2.ncbiobank.org/Index_en). There is a wide distribution of registered diseases, and the number of registered diseases exceeds 200 disease types (Figure 2C). The primary sample types are blood and DNA, with spinal fluid and frozen tissue samples also collected depending on the characteristics of the disease. Quality-related information for blood and DNA samples, including preanalytical information such as collection, processing, aliquoting, and storage times, as well as nucleic acid quality indicators, can be provided to users upon request. For tissue samples, the availability of quality-related information varies depending on the biobank and biospecimen. Users can search the catalogue database to identify how many and which types of samples are available. Information on how to use the database has been described elsewhere (Omae et al., 2022). Since the 2022 review, the NCBN Catalogue Database has continued to be enhanced with the addition of features such as exclusion searches and support for subclassification search of the 10th revision of the International Classification of Diseases (ICD-10). Protocols and standard operating procedures (SOPs) related to sample handling and quality control at the six NCBN biobanks are also publicly available on the NCBN website.

**FIGURE 2:**
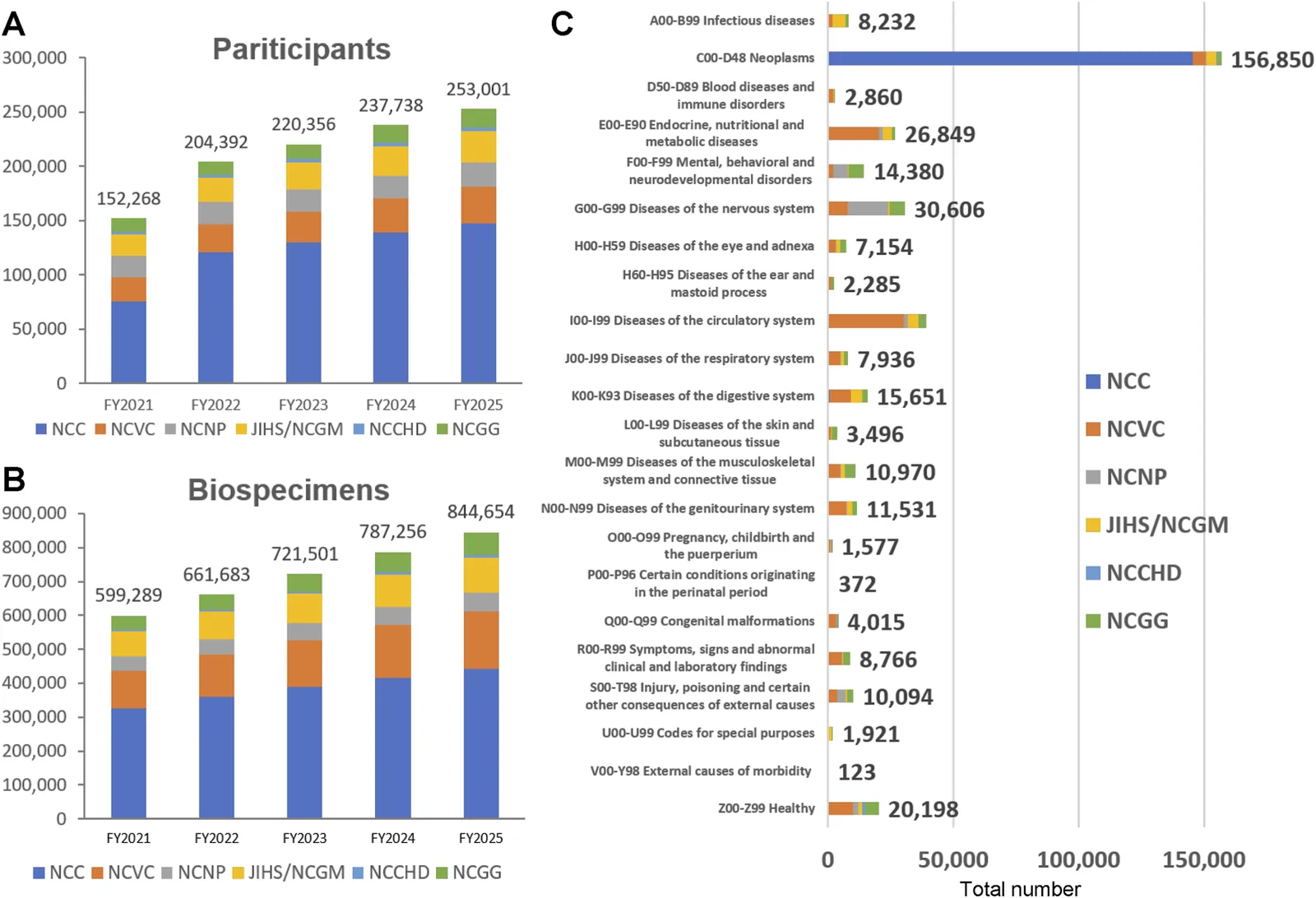
Overview of registered participants and biospecimens in the NCBN. **(A,B)** Cumulative number of registered participants and biospecimens over past 5 years. **(C)** Distribution of each disease classification in the ICD-10.

### 2.2 Overview of sample utilization

As the number of registered samples increases, the utilization of stored samples is also progressing. Table 1 shows the cumulative number of samples provided as of the end of March 2026. More than 200,000 human biospecimens accompanied by clinical information have been provided to companies and academic institutions (Table 1). The primary characteristic of the NCBN bioresources is the availability of diverse patient-derived samples linked to detailed, high-quality clinical information that have been collected by the physicians of each NCBN biobank. Users can choose between two usage models: collaborative research with institutions within the NCBN, and provision, which requires a fee covering actual costs but grants the user ownership of the intellectual property. As of our previous review (Omae et al., 2022), provision was available at 4 NCs; the NCC began offering this option in 2025, and the NCVC has discussed the process to start provision. At present, NCBN samples are available to overseas users through joint research with any of the biobanks within the NCBN. If a user belongs to a company, joint research between the company’s branch in Japan and the NCBN biobank is necessary. While consulting with other biobanks in Japan, efforts are currently underway to establish a system for providing samples to overseas institutions.

**TABLE 1:** Summary of sample provision by NCBN biobanks (as of March 2026).

NCs	Cumulative number of collaborative studies	Cumulative number of collaborative institutions			Cumulative number of samples provided (biospecimens/information)		Cumulative number of relevant publications
		Company	Academia	Other	Collaborative research	Provision	
NCC	594	367	503	268	57,226/0	0/0	1,334
NCVC	83	23	129	32	56,331/31,321	0/0	96
NCNP	130	31	143	117	23,998/4,769	5,012/674	367
JIHS/NCGM	150	23	174	77	27,993/34,850	7,929/0	171
NCCHD	89	1	96	6	2,200/591	964/2,872	194
NCGG	195	71	413	312	81,352/170,034	31,219/50,246	268
Total	1,241	516	1,458	812	249,100/241,565	45,124/53,792	2,430

### 2.3 Representative examples of the utilization of NCBN samples and medical records

Detailed clinical information is essential for conducting disease research. The value of a sample increases significantly depending on the richness and accuracy of this accompanying clinical information. As a reference for future use, we would like to introduce the key features of each NCBN biobank, as well as the representative studies to illustrate the unique strengths of each biobank.

The NCC Biobank is distinguished by its ability to conduct cancer analyses to generate drug discovery seeds as well as germline analyses to elucidate cancer susceptibility and treatment response, which form the foundation of personalized medicine. The NCC Biobank is the largest cancer biobank in Japan, and besides major cancers, such as gastric, lung, breast, and colon cancer, it has collected a large number of rare cancer cases, such as brain, ophthalmic, head and neck cancer, and bone and soft tissue sarcoma, which are difficult to collect at other institutions. As part of the International Cancer Genome Consortium, the results of whole-genome sequencing analysis of cancer samples from the NCC Biobank have been published. In clear cell renal cell carcinoma, underlying carcinogen exposures were inferred by sequencing the genomes of cancers from populations with different incidence rates. The detection of differences in somatic mutation profiles revealed a mutational signature of unknown cause in more than 70% of cases in Japan but in less than 2% elsewhere, indicating the existence of multiple, geographically variable, mutagenic exposures (Senkin et al., 2024).

The NCVC Biobank aims to provide a foundation for basic and clinical medical research that can contribute to overcoming major diseases, particularly cardiovascular diseases (including cardiomyopathy, arrhythmia, hyperlipidemia, heart failure, ischemic heart disease, pulmonary hypertension, and aortic aneurysm) and cerebrovascular diseases, through the centralized collection and management of these samples. As a signature sample set, the NCVC Biobank began collecting time-series samples from the same subjects at multiple time points-spanning from the hyperacute to the chronic phases-of ischemic heart disease, a major cardiovascular condition, in 2019. Analysis of NCVC Biobank genomic and clinical data, including echocardiographic data, revealed that a specific mutation in the *RNF213* gene, which is associated with the onset of many cases of moyamoya disease (a cerebrovascular disorder) and other diseases among Asians, may be linked to changes in cardiac-to-pulmonary blood flow and an increased risk of refractory pulmonary hypertension. This finding has contributed to the development of genetic testing that may lead to improved disease prevention and more effective treatment (Noda et al., 2025).

The NCNP Biobank aims to support research on overcoming psychiatric and neurological disorders, such as schizophrenia, major depression, Parkinson’s disease, epilepsy, and multiple sclerosis, by providing samples and information. The NCNP Biobank systematically collects and stores disease-relevant biospecimens, such as cerebrospinal fluid (CSF), brain tissue, muscle tissue, and blood, from both patients and healthy controls, with the help of dedicated medical staff. CSF contains numerous molecules reflecting physiological and pathological processes in the central nervous system. The NCNP Biobank’s CSF collection is among the largest and most extensively characterized disease-oriented CSF resources in Japan. Additionally, specialized clinical staff collect high-quality clinical information necessary for research. Basic research findings using induced pluripotent stem cells derived from patients with Alzheimer’s disease and healthy individuals suggest that an increase in BMP4 protein concentration in the cell culture supernatant leads to neurogenesis failure in Alzheimer’s disease, and additional analyses utilizing the CSF of Alzheimer’s patients and healthy individuals from the Biobank confirmed elevated BMP4 concentrations in patients with Alzheimer’s disease, thereby providing insights into the mechanisms underlying cognitive decline in such individuals (Nakatsu et al., 2023).

The JIHS Biobank aims to create synergy between basic and clinical research to advance medical science. The JIHS Biobank analyzes the biological and sociological factors underlying infectious diseases, such as AIDS, tuberculosis, malaria, and hepatitis, as well as emerging and reemerging infectious diseases. Furthermore, leveraging its status as the only general hospital within the NCBN, the JIHS integrates clinical care initiatives with research activities to treat complex patients with multiple comorbidities. This includes child mental illness, a field with few specialists both domestically and internationally. The JIHS has established biobanks at the NCGM in Shinjuku and the National Kohnodai Medical Center in Ichikawa, Chiba area, and the procedures for using these resources have been unified. Samples from the JIHS/NCGM Biobank, which specializes in infectious disease, were utilized as part of the international consortium called the COVID-19 Host Genetics Initiative, and the obtained genomic sequencing data were utilized for an exome-wide association study from 21 cohorts across 12 countries. Rare variant analyses of 5,085 severe COVID-19 cases and 571,737 controls revealed a rare deleterious variant in the SARS-CoV-2 sensor toll-like receptor *TLR7*, which increases the risk for severe COVID-19 by 5.3-fold, revealing similarities and differences across countries and genetic backgrounds, thereby providing additional insights into factors influencing COVID-19 outcomes (Butler-Laporte et al., 2022).

The NCCHD Biobank aims to establish a biobank capable of collecting samples related to pediatric and obstetric conditions, such as gestational diabetes, pregnancy in women, newborn babies, low birth weight, and particularly rare and intractable diseases. For cases involving fetal abnormalities and pregnancy complications, the NCCHD Biobank collects genomic DNA from two or three generations: the affected child (including the fetus), the parents, and the grandparents. While the collection extends beyond rare and intractable pediatric diseases to include cases of normal deliveries, all cases managed and delivered at the NCCHD Hospital constitute a bioresource (a Japanese cohort of “normal” pregnancy and delivery histories) supplemented with detailed information about the clinical course of the pregnancies. This makes it uniquely suitable as a control sample for a wide range of applications, particularly for the analysis of congenital disorders and perinatal abnormalities. The collection of rare diseases is made available to researchers to improve disease diagnosis and prognosis prediction. Whole-genome DNA methylation data obtained from patients with gonadotropin deficiency (a neuroendocrine disorder characterized primarily by external genital abnormalities, delayed puberty, and infertility) and unaffected controls in the NCCHD Biobank revealed a patient-specific DNA methylation change in the *ZNF254* gene promoter region, which may help to understand the pathogenesis of the disease (Suzuki et al., 2024).

The NCGG Biobank recruits, manages, and distributes biological samples and associated clinical data, focusing primarily on diseases common among older adults, such as Alzheimer’s dementia, mild cognitive impairment, dementia with Lewy bodies, lumbar spinal canal stenosis, and normal pressure hydrocephalus. The NCGG Biobank and Medical Genome Center actively facilitate large-scale genomic analysis of registrants from hospitals and have developed infrastructure for dementia research (integrated Database for Dementia Research (iDDR); https://www.ncgg.go.jp/ri/biobank/study/iddr.html), which is capable of conducting integrated analyses of clinical medical records, imaging data, and omics data. In collaborative research, the NCGG researchers conducted a genome-wide association study using genomic data from patients with dementia with grain (DG), Alzheimer’s disease, and cognitively healthy older adults, and identified a genomic locus showing an association with genome-wide significance for DG. Further analysis of expression quantitative trait loci by integrating gene expression data in the human brain revealed a statistically strong association with *DAPK2* gene expression in the frontal lobe, representing the first study to identify risk genes associated with DG, thereby conferring to the understanding of the pathology of DG and dementia, which presents complex clinical manifestations (Mitsumori et al., 2026).

### 2.4 Achievements in whole-genome sequencing of NCBN control samples

Whole-genome sequencing analysis of 9,850 NCBN samples was performed with the support of the Japan Agency for Medical Research and Development (AMED). We call the dataset “NCBN Control”, because the WGS project was conducted to establish control data for the national genomics initiative on cancer and rare disease in the Japanese population, and the sequenced samples were selected based on their ICD-10 codes. We first provided an overview and reported the characteristics of the genetic diversity of NCBN Control samples based on whole-genome sequencing data. The NCBN samples included the Ryukyuan population, which has a different genetic background to that of mainland Japanese (Kawai et al., 2023). Statistical information on genetic polymorphisms among NCBN Controls is publicly available in the NBDC Human Database (hum0331. v1) and MGeND (Kamada et al., 2019), and individual genome data have also been shared under 28 collaborative research studies with the approval of our Data Use Review Committee and contribution as NCBN Controls WGS Consortium. Data provision is mainly conducted by the AMED Genome group sharing Database (agd0023. v1). To date, nine research papers resulting from collaborative research that use these data as non‐cancer controls, for variant filtering and as an imputation reference panel for each study, have been published (Table 2) (Shimoda et al., 2023; Nakamura et al., 2024; Naito et al., 2024; Imai et al., 2024; Suga et al., 2024; Orimo et al., 2024; Yazaki et al., 2025a; Cho et al., 2025; Yazaki et al., 2025b). The availability of genomic information for each sample is already searchable in the NCBN catalogue database.

**TABLE 2:** List of research utilizing the NCBN WGS controls dataset.

Study	Journal	Purpose
Kawai et al., 2023	PLOS Genet.	First publication reporting characteristics
Shimoda et al., 2023	Pharmacogenomics J.	Imputation reference panel
Nakamura et al., 2024	NPJ Genom Med.	Imputation reference panel
Naito et al., 2024	Inflamm Bowel Dis.	Imputation reference panel
Imai et al., 2024	Hum Genome Var.	Variant filtering
Suga et al., 2024	Genet Med Open.	Variant filtering
Orimo et al., 2024	J Hum Genet.	Non-neurodegenerative control
Yazaki et al., 2025a	EBioMedicine.	Non-cancer control
Cho et al., 2025	J Thorac Oncol.	Non-cancer control
Yazaki et al., 2025b	NPJ Breast Cancer.	Non-cancer control

## 3 Conclusion

The NCBN began operations in 2011 and has grown steadily to become Japan’s leading “disease biobank” in terms of both quality and quantity. The present review has provided an outline of the following characteristics of the NCBN: 1) it provides a diverse range of biological samples accompanied by accurate and detailed medical records based on the specific diseases each biobank specializes in; 2) a centralized catalogue database is available for searching samples; 3) more than 250,000 participants (mostly patients) and 800,000 biospecimens are registered in the network, and these numbers are growing; 4) more than 200,000 human biospecimens accompanied by clinical information have been provided to companies and academic institutions; and 5) some samples have undergone genomic analysis, and their utilization is currently underway. Together with other major Japanese biobanks, such as Biobank Japan (BBJ) and the Tohoku Medical Megabank (ToMMo), the NCBN contributes to a comprehensive national research infrastructure through an “All-Japan” framework called Biobank Network Japan. While BBJ and ToMMo provide valuable large-scale genomic and population resources, NCBN offers unique access to deeply phenotyped patients, rare diseases, specialized bioresources, and longitudinal clinical information via its disease-based biobank network. This complementary role positions NCBN as a vital platform for advancing translational research, biomarker discovery, and precision medicine in Japan.
